# Weighing the relative potential impacts of climate change and land‐use change on an endangered bird

**DOI:** 10.1002/ece3.2204

**Published:** 2016-06-08

**Authors:** Betsy A. Bancroft, Joshua J. Lawler, Nathan H. Schumaker

**Affiliations:** ^1^ School of Environmental and Forest SciencesUniversity of WashingtonSeattleWashington; ^2^ Western DivisionUnited States Environmental Protection AgencyCorvallisOregon; ^3^ Present address: Departments of Biology and Environmental StudiesGonzaga UniversitySpokaneWashington

**Keywords:** Development, environmental stress, habitat change, habitat loss, HexSim, *Picoides borealis*, population model, precipitation, Red‐cockaded Woodpecker, spatially explicit individual‐based model

## Abstract

Climate change and land‐use change are projected to be the two greatest drivers of biodiversity loss over the coming century. Land‐use change has resulted in extensive habitat loss for many species. Likewise, climate change has affected many species resulting in range shifts, changes in phenology, and altered interactions. We used a spatially explicit, individual‐based model to explore the effects of land‐use change and climate change on a population of the endangered Red‐cockaded Woodpecker (RCW;* Picoides borealis*). We modeled the effects of land‐use change using multiple scenarios representing different spatial arrangements of new training areas for troops across Fort Benning. We used projected climate‐driven changes in habitat and changes in reproductive output to explore the potential effects of climate change. We summarized potential changes in habitat based on the output of the dynamic vegetation model LPJ‐GUESS, run for multiple climate change scenarios through the year 2100. We projected potential changes in reproduction based on an empirical relationship between spring precipitation and the mean number of successful fledglings produced per nest attempt. As modeled in our study, climate change had virtually no effect on the RCW population. Conversely, simulated effects of land‐use change resulted in the loss of up to 28 breeding pairs by 2100. However, the simulated impacts of development depended on where the development occurred and could be completely avoided if the new training areas were placed in poor‐quality habitat. Our results demonstrate the flexibility inherent in many systems that allows seemingly incompatible human land uses, such as development, and conservation actions to exist side by side.

## Introduction

Climate change and land‐use change are likely to be two of the most important drivers of environmental change over the coming century (Sala et al. 2000). The best‐documented changes in biota in response to climate change are shifts in species distributions and changes in phenology. However, for many animal species, changes in climate are likely to lead to changes in habitat, food availability, and altered predator–prey and competitive relationships (Schneider et al. 2007). Species with specialized habitat requirements, narrow environmental tolerances, specific environmental triggers for phenological events, dependencies on species interactions, and poor dispersal abilities may be more susceptible to climatic changes (Foden et al. 2008).

Much of the Earth's surface has already been converted from its natural state for human uses (Kareiva et al. 2007), including the conversion of over 50% of the Earth's land surface for food production (Millenium Ecosystem Assessment 2005). Habitat loss and fragmentation (driven by land‐use change) have been the leading causes of species loss and decline over the last century (Wilcove et al. 1998; Carlson 2000; Lawler et al. 2002; Dirzo and Raven 2003; Stuart et al. 2004). More specifically, habitat loss affects many aspects of biodiversity, including species richness (Findlay and Houlahan 1997), population abundance and distribution (Schmiegelow and Mönkkönen 2002), genetic diversity (Lindsay et al. 2008), and population growth (Harper et al. 2008). However, changes in land use can result in habitat destruction or habitat creation, and the effects of land‐use change can vary by species and by the nature of the change. Thus, habitat loss can reduce the likelihood of population persistence for species with specialized habitat or diet requirements.

Together, climate change and land‐use change have the potential to significantly alter ecosystems, communities, and populations. Moreover, these two global changes can interact, resulting in greater alterations and positive feedback (Riordan and Rundel 2014). At large spatial scales, climate change and land‐use change have negative effects on species including birds (Barbet‐Massin et al. 2012; Kampichler et al. 2012). Despite these large‐scale trends, climate change and land‐use change may influence populations and species differently, such that some populations may be primarily threatened by climate change, while others are more susceptible to land‐use change. For managers, understanding which threat is preeminent for a given population, in addition to understanding the potential interaction among stressors, is vital to creating successful conservation plans. Here, we explore how both climate change and land‐use change may influence an endemic, endangered US species.

The Red‐cockaded Woodpecker (RCW, *Picoides borealis*) was listed as an endangered species in 1970, largely due to widespread habitat loss (US Fish and Wildlife Service 2003). RCWs are dependent on mature, open, pine woodlands maintained by frequent fire. These pine ecosystems have suffered widespread declines due to logging for timber and agriculture, coupled with fire suppression (US Fish and Wildlife Service 2003). The majority of remaining RCW populations are found on federal lands, and consequently, the Red‐cockaded Woodpecker Recovery Plan identifies national forests and military installations as critical for recovery (US Fish and Wildlife Service 2003). Although US military installations provide sanctuary for many threatened and endangered species, training of military troops frequently requires intensive alteration of the landscape, often in ways that are not compatible with habitat and foraging needs of the various species living on the installation. Furthermore, these installations will be as susceptible to the impacts of climate change as other parts of the landscape. Climate‐driven changes in the pine forests of the southeastern USA have the potential to eliminate what remains of suitable RCW habitat (Hansen et al. 2001). Furthermore, changes in precipitation could alter insect availability or foraging behavior. Thus, this RCW population is likely threatened by both climate change and land‐use change despite their protected status as a listed species.

Here, we use a spatially explicit, individual‐based population model to explore the relative effects of climate change and land‐use change on the RCW population on Fort Benning, located near Columbus, Georgia, USA. Fort Benning has fewer than 300 RCW clusters – RCWs breed in small cooperative groups that use a cluster of cavity trees – and is designated as a primary core recovery population (US Fish and Wildlife Service 2003). The installation's recovery goal is 351 potential breeding pairs across the approximately 74,000‐ha installation. The installation is dominated by loblolly pine (*Pinus taeda*), but historically was likely dominated by longleaf pine (*P. palustris*). Forestry practices have varied since the founding of the installation (reviewed in Doresky et al. 2003); however, current forest management includes prescribed burns on an approximate 3‐year cycle and planting longleaf pine seedlings. We compare the impacts of multiple spatial arrangements of land‐use change, climate‐driven changes in vegetation, and the potential for climate‐driven changes in reproduction on population dynamics over a 100‐year period.

## Methods

### Habitat model

We used random forests (Breiman 2001), an ensemble classification technique, to build predictive models of habitat probability using data on forest composition and structure at Fort Benning, GA (Table 1). We divided known presence and absence data into training and test data sets by randomly withholding one‐third of presences and one‐third of absences (90 points each) to serve as test data. The remaining data were used to train the models. We used the R statistical package RandomForest to build model predictors. For each model, we built 500 trees using two randomly selected variables of the three total predictor variables as candidates for each split in the tree. This habitat map correctly identified active clusters in the test data 81% of the time and correctly identified absences in the test data 88% of the time. Although including more parameters may have increased the overall accuracy of the habitat map, all parameters in the model could be projected under future conditions. This baseline habitat map was the foundation for all further habitat maps generated under the scenarios described below. This map and all other spatial layers were developed on a grid of 50‐m × 50‐m cells.

**Table 1:** Environmental variables used in constructing Red‐cockaded Woodpecker habitat probability models. Data were derived from forest inventory data collected in 2003 at Fort Benning, Georgia, USA

Variable	Type	Range of values
Average age^a^ of trees within stand is greater than or equal to 30, or less than 30 years old	Categorical	≥30 <30
Average age^a^ of trees within stand is greater than or equal to 60, or less than 60 years old	Categorical	≥60 <60
The functional type of the dominant species in the stand	Categorical	Conifer Hardwood

a Age parameters were adjusted to reflect current age of stands.

### Simulating land‐use change

**Figure 1:**
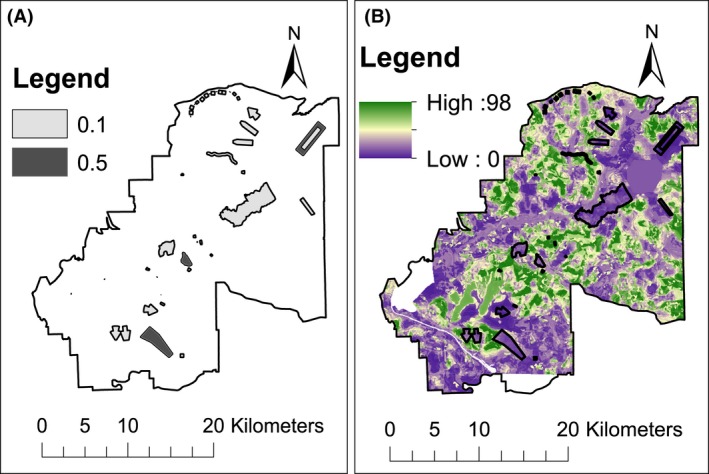
(A) Map of training ranges added to the landscape at Fort Benning. This map formed the baseline range development scenario from which all other range development scenarios were generated. Values indicate the effect of each range on Red‐cockaded Woodpeckers (RCW). The habitat ratings in the initial habitat probability map were multiplied by the values shown to downgrade habitat quality where new ranges were placed. Lower values indicate a larger negative effect of the range on RCW habitat. (B) Baseline development habitat map for RCW using proposed training ranges shown in A. Each range shown in A was placed on the landscape in the location shown in A and habitat within the range was reduced based on estimated impact of the range on Red‐cockaded Woodpecker habitat quality. Values in the legend represent likelihood of RCW presence, where higher values indicate higher likelihood of RCW presence.

At military installations such as Fort Benning, land‐use change occurs primarily through the development of training areas. To simulate land‐use change, a map of proposed training areas was used as a baseline of future development (Fig. 1A). These areas do not necessarily represent actual development on the installation, but are useful to estimate the land area that might change due to additional training needs. The training areas range in size from 0.02 ha to 1107.98 ha (median: 9.00). The training areas shown in Figure 1A were added to the baseline landscape by reducing the habitat quality within each area according to the estimated impact of each development on RCW habitat (M. Barron, pers. comm.). For example, areas designated as buffer zones around firing ranges had less impact (habitat quality reduced by 50%) on habitat than did the large area that was to be clear‐cut and used for tank training (habitat quality reduced by 90%). Each pixel within each development was multiplied by the value shown in Figure 1A, such that a development with a rating of 0.1 would reduce the existing habitat to 10% of the original habitat probability. This new habitat map with the developed areas in place, hereafter “baseline development map” (Fig. 1B), reflects a current plan for base development, and thus, land‐use change to which the base is already largely committed. We developed three development scenarios that built off of this baseline development map. In these three development scenarios, three or four new areas were developed every 10 years during the 100‐year simulations. The area of additional development in the scenarios is equal to the area shown in Figure 1A, and thus, by the end of the 100 years, the area of new development will be twice that shown in Figure 1A (total new development equal to 8.6% of the land area).

We generated three development scenarios (Conservation, Convenience, and Worst‐case) with three replicate landscapes for each (Table 2). In the Conservation scenario, new developments were placed in poor‐quality habitat (below 0.57, Table 2). In the Convenience scenario, developments were placed within 400 m of a road. In the Worst‐case scenarios, developments were all placed within RCW habitat. For each scenario, developments (drawn at random without replacement from the set of development areas depicted in Fig. 1A) were placed randomly within constraints listed above (which varied by scenario, Table 2) and habitat quality was reduced accordingly. Three replicate landscapes were generated for each of the three scenarios with a different random placement of the development areas. Maps were generated for each 10‐year increment, reflecting that decade's new developments, plus any previous decade's developments so that by year 2090, all new areas were in place on the final map for each scenario. All mapping and scenario building was completed in ArcGIS 9.3.1 (ESRI 2009).

**Table 2:** Description of land‐use change scenarios used to generate alternate future landscapes for Fort Benning, GA

Scenario class	Goal	Rules
Conservation	Minimize land‐use change in RCW habitat	The habitat model was converted into a binary model (habitat/not habitat) using the MaxKappa function in the PresenceAbsence package in R (Freeman and Moisen 2008). Habitat below the threshold (0.57) was designated as “non‐RCW habitat” and available for development
Convenience	Land‐use change based on location of roads on the installation	Using ArcMap 9.3 (ESRI 2009), current roads on Fort Benning were buffered with a 400‐m buffer. This buffer area was available for development
Worst‐case	Land‐use change only in RCW habitat	Habitat above the probability threshold calculated using MaxKappa (as above in Conservation scenarios) was designated as “RCW habitat” and available for development

### Simulating climate change

The potential effects of climate change on the RCW population at Fort Benning were assessed in two ways. First, we assessed the effect of future climate change on vegetation using the outputs of an ecosystem model, LPJ‐GUESS (ver. 030124; Smith et al. 2001). Vegetation projections were run for nine different future projected climates from three general circulation models (GCMs): CCSM3 (Collins et al. 2006), CGCM3.1(T47) (Scinocca et al. 2008), and UKMO‐HadCM3 (Pope et al. 2000) run for three emissions scenarios (B1, A1B, and A2 (Nakicenovic et al. 2000) (Shafer et al. 2012)). LPJ‐GUESS simulates vegetation in the form of plant functional types, such as broad‐leaved evergreens or grasses. These plant functional types can be combined to represent major biomes and habitat types (e.g., forest, grassland). LPJ‐GUESS also simulates changes in the fire regime and response of the vegetation to that change and to increased atmospheric CO_2_ concentrations. Following an 800‐year spin‐up period, LPJ‐GUESS was run for the years 1901–2100.

Second, future climate projections were used to model the potential effects of precipitation on RCW reproduction. Previous work suggested that spring precipitation may influence reproductive output in this species, primarily through reduced foraging activity by adults in heavy rain (Neal et al. 1993). In the current population, a significant negative relationship between RCW reproductive success (mean fledglings produced per nest attempt) and April precipitation was detected (*R* ^2^ = 0.62, *P *<* *0.01, *y* = 1.819 − 0.0024 × mean April precipitation). This relationship was used to project the effects of future climate on RCW reproduction. We extracted April precipitation from the nine future climate projections (Shafer et al. 2012) and mapped those projections for each year. The precipitation projections in these layers were then used to influence reproduction in the population model using the empirical relationship described above.

### Population simulations in HexSim

We used the HexSim modeling platform (version 1.4; Schumaker 2015) to build a population model for simulating the impacts of land‐use change and climate change on the RCW population at Fort Benning. HexSim is a spatially explicit individual‐based modeling framework that simulates population dynamics over time given spatial data layers and population parameters. HexSim is a framework in which simulation models are constructed. HexSim itself contains no embedded parameters, equations, or specific processes that must be incorporated into its models. HexSim models are normally designed around available data, with poorly understood processes being left out, represented probabilistically, or explored via sensitivity analysis.

**Figure 2:**
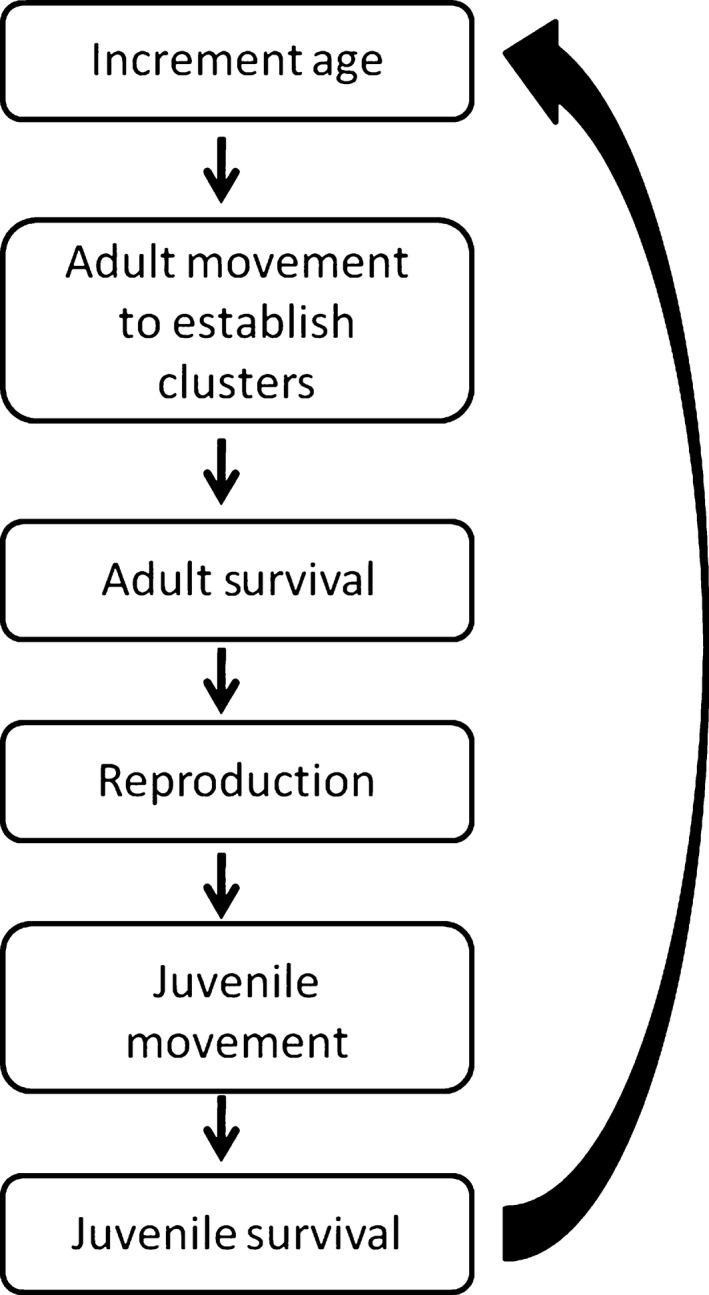
Red‐cockaded Woodpecker annual cycle as implemented in HexSim. At the beginning of each year, 1 year was added to the age of each individual present in the landscape (“increment age”). All movement routines, survival, and reproductive rates were modeled using the values described in the text and Table 3. In these scenarios, “adults” are individuals surviving the first year.

The population model followed the yearly cycle of events typical for RCWs (Fig. 2). HexSim was parameterized using data from the literature (Walters et al. 1988; Walters 1990; DeLotelle and Epting 1992; LaBranche and Walters 1994; Engstrom and Sanders 1997; Conner et al. 1999, 2004; Daniels and Walters 2000; Doresky et al. 2001; Leonard et al. 2003). Although HexSim makes constructing extremely complex models possible, we attempted to build the simplest defensible model that captures the essential aspects of RCW biology. As reproductive output for a population of RCW is limited by the number of females of reproductive age (Conner et al. 2010), we modeled females only. Females are rarely helpers in cooperatively breeding RCW clusters; thus, the helper class was not modeled (Conner et al. 2010). Females without established clusters were modeled as a separate floater class. This floater class moved through the landscape and settled available territories vacated after the death of the previous female, or areas that were not part of another female's territory. Floaters did not reproduce.

We parameterized HexSim to start with 800 RCWs (ages 0–14 years) placed randomly in the landscape. This number was selected to be far above the potential carrying capacity of the population to ensure that the simulated woodpeckers had an opportunity to become established throughout the landscape. After a 50‐year initialization period, the model stabilized at approximately 290 RCW breeding pairs on the landscape in the baseline scenario, which is similar to the current number of RCW pairs present at Fort Benning (~280 breeding pairs).

The maximum age for any RCW on the landscape was 14 years, which when coupled with the adult survival rate (74% for adults on a territory, 25% for adult floaters), allowed for birds older than 10 years in the landscape, but birds in these older age classes were relatively rare. Fledgling survival was set at 45% (Walters et al. 1988; Walters 1990).

We parameterized the model so that quality of a cluster was based on total habitat quality (sum of all hexagons in the cluster). Target quality for establishing a cluster was set such that the spatial occupancy of the modeled population reflected current spacing and density of RCWs at Fort Benning. Any hexagon with a habitat quality of at least 1% of maximum was eligible for inclusion in RCW home ranges. Thus, RCW home ranges could include areas of low‐quality habitat, including fragmented areas, provided enough high‐quality habitat was present to meet the minimum range value. In addition, home ranges in our model can vary in size widely based on quality of available habitat, allowing for higher density in areas of high‐quality habitat. Any RCW that did not find a home range that met the minimum range quality became a floater without a home range. These floaters would move again in the next yearly cycle to try to establish a home range, following the movement routine (Table 3). As young female RCW do not typically stay in their natal territory (Conner et al. 2010), all juvenile birds (fledglings) became floaters in their first year and moved to establish a new home range.

**Table 3:** Parameters used to model adult and fledgling Red‐cockaded Woodpeckers at Fort Benning in HexSim

Category	Parameter	Value
Reproduction	Expected rate	1.61 offspring/cluster (range 0–6)
Territory assessment	Range dynamics	Improve deficient ranges
Floater creation	% Resource target	Float if cluster resources are <90% of target
Adult movement	Strategy	Exploration^a^ then dispersal
	Maximum number of explorations	2
	Maximum explored area	500 hexagons, adaptive^b^
	Mean path lengths for dispersal (log normal distribution, in hexagons)	10 + 5 SD
	Path length bounds	1–1000 hexagons
	Stopping criteria	Stop dispersal if mean resource quality of 60 is experienced over 100 hexagons
Fledgling movement	Strategy	Exploration then dispersal
	Maximum number of explorations	3
	Maximum explored area	800 hexagons, adaptive
	Mean path lengths for dispersal (log normal distribution, in hexagons)	130 + 10 SD
	Path length bounds	10–1000 hexagons
	Stopping criteria	None

a Exploration simulates an intensive search for resources in a surrounding area.

b Adaptive strategy simulates limited knowledge of surroundings in RCW.

Yearly spatial RCW population data do not exist for this population, so we were unable to compare modeled and actual population distribution as a method of model evaluation (i.e., hindcasting). We ran the model 20 times for each replicate development scenario (nine different series of landscapes) and climate change scenarios (nine different climate scenarios). The spatial data layers (described above) were incorporated into the simulations at 10‐year (land‐use) or 1‐year (climate) intervals to explore the effects of two factors on the RCW population.

### Statistical analysis

Final population size (number of potential breeding pairs) was compared among the scenario classes (*n* = three per class, 20 replicate simulations per scenario) or climate scenario (*n *=* *20 per climate scenario) using analysis of variance (ANOVA), followed by a post hoc test for least significant differences using a Tukey's HSD test. Data were assessed for normality prior to analysis using normal Q–Q plots. All analyses were conducted in R version 2.13.0 (R Core Team 2011).

## Results

**Figure 3:**
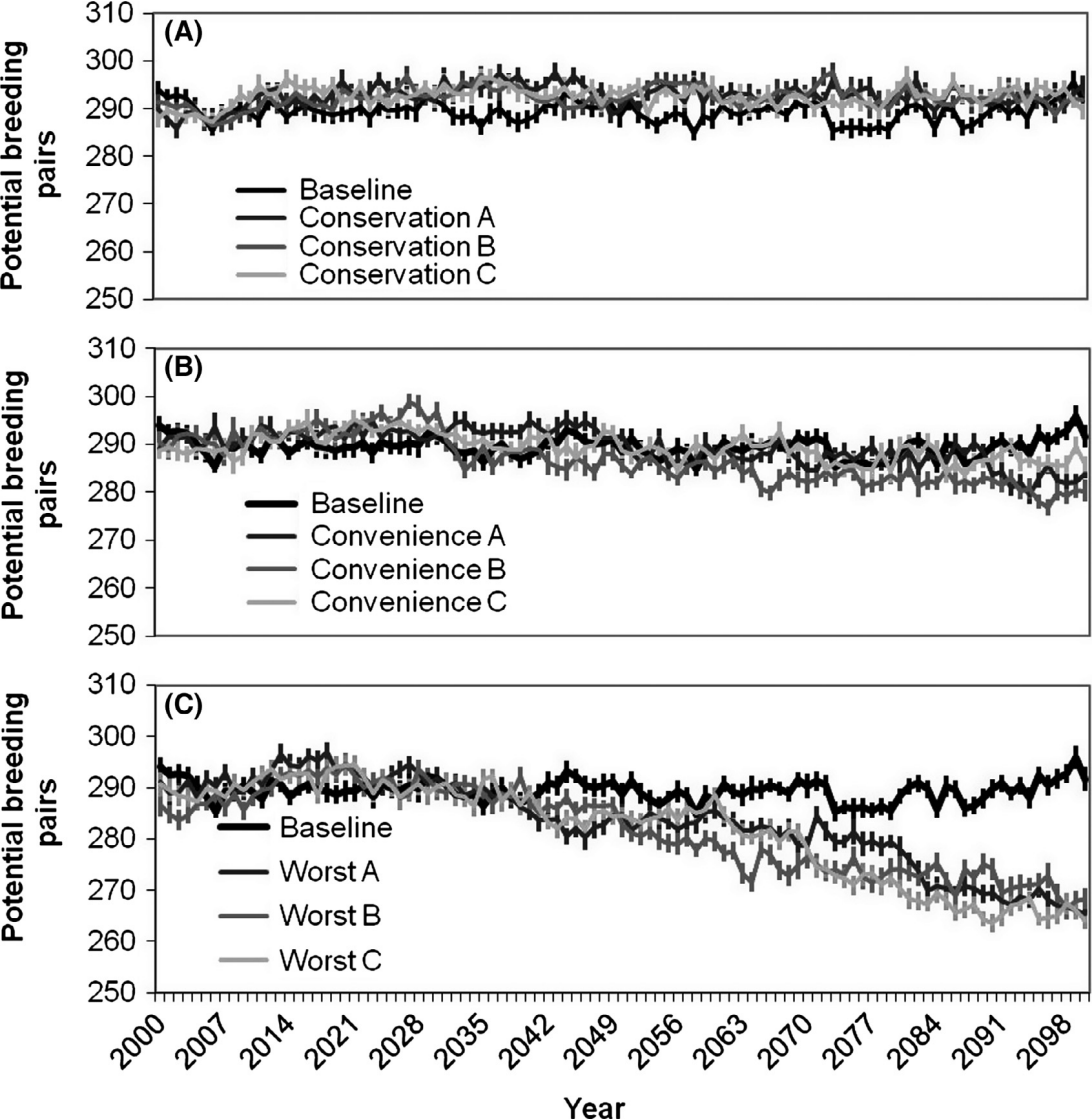
Red‐cockaded Woodpecker (RCW) population projections under three scenarios and baseline conditions. Lines represent means of 20 replicate model runs plus 1 standard error. (A) Conservation land‐use scenarios. RCW population projections are not different from baseline development (no further development) in all Conservation scenarios. (B) Convenience land‐use scenarios. Population projections are slightly lower than baseline development simulations (no further development) in all Convenience scenarios. (C) Worst‐case land‐use scenarios. Population projections are much lower than baseline (no further development) in all Worst‐case scenarios.

The effects of development of new training areas on the RCW population strongly depended on which development scenario was applied (Fig. 3, *P *<* *0.001, *F*_2,8_ = 82.8). Under the Conservation scenario, the population was relatively stable over the 100‐year simulation period (Fig. 3A). Conversely, the population declined slightly under the Convenience scenario (Fig. 3B) and declined strongly under the Worst‐case scenario (Fig. 3C). By the year 2100, the RCW population was on average 26 breeding pairs (9%) smaller in the Worst‐case scenario than in the Conservation scenario. The final population size was different among the three development scenarios (*P *<* *0.05 for all pairwise comparisons).

No changes in the dominant vegetation were projected for Fort Benning under any climate model or emissions scenario. The LPJ‐GUESS model outputs suggested that the study area would remain suitable for needle‐leaved evergreen trees, the primary habitat type of RCW. Thus, as we modeled it, climate change had no impact on RCW habitat at Fort Benning. Similarly, our simulations showed little impact of changes in April precipitation on the final number of potential RCW breeding pairs at Fort Benning. Population trends resulting from the simulations using the projected future climates were not different from those resulting from simulations run with a constant (current) climate (*P *>* *0.05).

## Discussion

Our findings suggest that habitat loss (modeled here through land‐use change) may be a more important threat to some species than climate change. Similar patterns have been found for birds at a global scale (Jetz et al. 2007) and several other organisms. For example, a recent study found that high‐elevation Andean plant species were highly susceptible to human land‐use change, while climate change in the absence of land‐use change had a smaller negative effect on these plants (Feeley and Silman 2010). Similarly, extinction risk for three Mexican cloud forest species was low despite projected climate change when the remaining cloud forest was protected from land‐use change, largely because dispersal was sufficient to prevent extinction when the landscape was relatively intact (Ponce‐Reyes et al. 2013). Thus, RCW, like many other species, may be more susceptible to land‐use change than climate change.

Our finding of a strong effect of a reduction of habitat quality for RCW in high‐quality areas is not particularly surprising, given this species' very specific habitat requirements. RCWs are dependent on mature pine savanna habitat, particularly longleaf pine systems (Walters 1990). Indeed, declines in this species have been attributed primarily to habitat loss (US Fish and Wildlife Service 2003). Some evidence suggests that RCW home ranges tend to be smaller when they include high‐quality habitat (Engstrom and Sanders 1997; Convery and Walters 2003). Thus, areas with higher quality habitat could support more RCW clusters. These studies combined with the results of the current study suggest that providing more high‐quality habitat will help increase the number of RCW on the landscape in areas that are not developed.

Even in the baseline development scenario, which included only planned developments that have either already occurred or are slated to occur in the near future, the installation did not meet its recovery goal of 351 breeding pairs, suggesting that without additional management, the installation will not meet the recovery goal. Most of this observed population growth on Fort Benning in the past several years was likely driven by successful cavity creation (Doresky et al. 2003). However, it remains to be seen whether artificial cavity creation can sustain population growth in the face of landscape changes that include new development for training. Indeed, the United States Fish and Wildlife Service issued a Biological Opinion stating that the planned land‐use changes used as our baseline development scenario would jeopardize RCW recovery (US Fish and Wildlife Service 2009).

Climate change had little effect on RCW habitat in our ecosystem models. Although other broad‐scale studies have projected potential changes for the vegetation of much of the southwestern USA, the vegetation projections that we used showed little change in the general type of forest at Fort Benning over the coming century (Shafer et al. 2012). While these models give some idea of the basic vegetation types that will dominate an area, they do not provide projections of which plant species will dominate or whether the structure of the forest will change in more subtle ways. Although our models did not indicate a change in forest cover, if climate change results in an increase in the hardwood midstory, RCW may be negatively affected. Presence of hardwood midstory vegetation is associated with cluster abandonment in RCW and low habitat suitability (Conner et al. 2010). Thus, increases in hardwood midstory would either reduce the habitat suitability for RCW, or require expensive manual removal of hardwoods from RCW habitat.

Changes in precipitation due to climate change did not result in reduced numbers of potential RCW breeding pairs. This result was surprising given the projected increase in spring precipitation (range: +1% to +27%) for the Fort Benning study area (Shafer et al. 2012). However, precipitation as high as 300 mm for the month of April did not result in mortality of all fledglings within a cluster, suggesting that although high levels of precipitation may reduce the overall number of fledglings that successfully fledge, each cluster is still able to produce at least one fledgling. Because each cluster is still reproducing, it is possible that the population as a whole is still buffered from years of low reproduction due to the floater class of older but unreproductive females. The results suggest that RCW may be less vulnerable to climate change than other species because of the floater class, which also buffers RCW populations against demographic stochasticity (Walters et al. 2002). Furthermore, evidence suggests that RCW may respond to changes in climate by laying eggs earlier (Schiegg et al. 2002), suggesting that RCW may be phenotypically plastic in response to some aspects of climate change.

Although we did not observe any effects of climate change on RCW as simulated here, it is important to note that our study only addressed two potential ways in which climate change could affect the population. The empirical relationship between April precipitation and reproduction from Ft. Benning that we used in our simulations has been noted elsewhere and it is hypothesized that it is driven by decreases in the number of foraging bouts by adults (Neal et al. 1993). If the overall increase in precipitation projected for the area reduces the number of foraging bouts such that individual body condition declines, mortality rates in adults or young may increase in addition to the decrease in fecundity that has been observed. There are myriad potential effects that climate change may have on this and other species. For example, climate change may affect the population dynamics of cavity competitors, diseases, and parasites, or the distribution or abundance of nest predators. The interaction among insects and plants may also be influenced by climate change (DeLucia et al. 2012). If insect abundance decreases or the timing of insect population growth (i.e., larval development for many species) changes as a result of climate perturbations, then food availability for animals such as the RCW may be negatively influenced. Furthermore, our models did not include the well‐documented phenomenon of loblolly pine decline observed at Fort Benning. In a recent study, loblolly decline was related to water stress (Ryu et al. 2013), suggesting that loblolly pines might be more susceptible to decline under changing climates. Thus, climate change should not be discounted as a threat to this species, and future work should focus on how climate change will influence the entire ecosystem upon which this species depends.

Our findings suggest that prioritizing habitat conservation may enhance the resilience of many systems to climate change. A recent analysis found that for temperate deciduous forest and warm mixed forest biomes, land conversion due to agriculture and timber expansion is expected to have a much larger effect on biodiversity loss than climate change, while the tundra, savannas, boreal forest, and cool conifer biomes are expected to be more impacted by climate change (Sala et al. 2005). Targeting conservation actions by identifying which systems are primarily threatened by each stressor could increase the return on investment for conservation dollars, as land‐use change could be restricted in those areas highly susceptible to development and those systems where dispersal through intact habitat may counteract the negative effects of climate change, while climate adaptation and management activities could focus on those ecosystems with higher susceptibility to climate perturbations.

Our results stress the importance of land‐use change even in the face of large projected shifts in environmental conditions, such as those associated with climate change. Perhaps more importantly, although tempered by the caveats above, this is a positive message. Despite the fact that land‐use change is a formidable threat that has already caused the extinction of many species, it is a threat for which there are clear solutions. Climate change is a far less manageable challenge, many of the true solutions to which will require coordinated action on a global scale.

## Conflict of Interest

None declared.
